# Facilitating Novice Visual Search with tES over rIFG: Baseline-Dependent Gains in Target Identification

**DOI:** 10.3390/brainsci16010001

**Published:** 2025-12-19

**Authors:** Bradley M. Robert, Aaron Winder, Mason S. Briggs, Gabriella I. Atencio, Vincent P. Clark

**Affiliations:** 1 Psychology Clinical Neuroscience Center, Department of Psychology, University of New Mexico, Albuquerque, NM 87131, USA; 2 Charles River Analytics, Cambridge, MA 02138, USA

**Keywords:** transcranial direct current stimulation, transcranial random noise stimulation, visual attention, synthetic aperture radar, target identification, change detection, baseline performance, right inferior frontal gyrus, cognitive enhancement

## Abstract

**Background/Objectives:** Transcranial electrical stimulation (tES) shows potential for enhancing attention and learning, yet its effects in applied contexts remain underexplored. This study investigated whether transcranial direct current stimulation (tDCS) either alone or in combination with high-frequency transcranial random noise stimulation (hf-tRNS) over the right inferior frontal gyrus (rIFG) could enhance performance in a visual search task requiring target identification and change detection, compared with a low-current control condition. **Methods:** Sixty-four participants were randomly assigned to receive tDCS alone (2.0 mA), tDCS with hf-tRNS (1.8 mA DC offset combined with 100–500 Hz noise at ±0.18 mA), or low-current control stimulation during training. The task involved identifying vehicles and detecting changes between image presentations. Performance accuracy and EEG oscillatory power were assessed at baseline and post-stimulation. **Results:** ANCOVA revealed significant effects of stimulation on target identification accuracy (F(2,60) = 3.27, *p* = 0.045, η*p*^2^ = 0.098), with tDCS showing greater improvement than the low-current control condition (*p* = 0.017). No significant effects were found for change detection for any stimulation condition, or for either the target or change detection for hf-tRNS. Baseline performance moderated stimulation effects: low performers receiving tDCS showed the greatest improvements (F(2,26) = 3.80, *p* = 0.036, η*p*^2^ = 0.226), surpassing even high-baseline performers post-training. EEG analyses revealed that participants who showed greater decreases in frontal theta power demonstrated larger improvements in accuracy with tDCS alone (r = −0.634, *p* = 0.005) but not with hf-tRNS or the control. **Conclusions:** tDCS over rIFG selectively enhanced target identification accuracy in a complex visual search, particularly benefiting individuals with lower-baseline performance. These findings suggest tDCS may facilitate training in lower-performing novice populations.

## 1. Introduction

Visual search, a fundamental cognitive process enabling identification of task-relevant stimuli within complex environments, underlies diverse behaviors from basic object recognition to specialized tasks like radiological image interpretation [1,2]. Efficient visual search depends on the interaction between bottom-up (stimulus-driven) and top-down (goal-directed) attention mechanisms [3]. At the neural level, this process engages distributed cortical regions including the intraparietal sulcus (IPS), frontal eye fields (FEF), and dorsolateral prefrontal cortex (DLPFC), organized into dorsal and ventral attention networks [4,5].

In applied settings requiring sustained vigilance, such as driving, piloting, air traffic control, and intelligence analysis, maintaining efficient visual search over extended periods is critical. The well-documented “vigilance decrement” reflects the decline of performance over time, highlighting the challenge of sustaining attention for long periods [6]. These demands are particularly relevant in military contexts, where analysts must detect targets and identify changes in complex synthetic aperture radar (SAR) imagery.

Transcranial electrical stimulation (tES) has emerged as a potential method for enhancing cognitive performance, including attention and visual search. Two tES approaches—transcranial direct current stimulation (tDCS) and transcranial random noise stimulation (tRNS)—operate through distinct mechanisms. tDCS applies constant current to modulate neuronal excitability [7], while tRNS uses stochastic fluctuations potentially enhancing signal detection through stochastic resonance [8,9]. Previous research in our laboratory and that of others has demonstrated tDCS-related improvements in visual attention tasks [10,11], with effects often dependent on baseline performance levels [12,13]. Other studies have shown tRNS applied during training can enhance learning and post-training performance on perceptual and complex cognitive tasks, improving neuroplasticity in visual perceptual learning and boosting training efficacy in both healthy adults and clinical populations [14,15]. In our previous studies, we have observed that some devices used for producing tDCS current include high-frequency transients (due to specific characteristics of their electrical design) in addition to the desired DC current, and that these appear to produce larger behavioral effects than “cleaner” tDCS systems [16]. This is further supported by prior studies that have shown that hf-tRNS can produce positive effects on behavior [17,18].

The present study investigated whether different tES protocols could enhance performance on a SAR visual search task in novice participants. A low-noise tDCS system was used to compare tDCS alone vs. tDCS combined with hf-tRNS. We targeted the right inferior frontal gyrus (rIFG), a key node in the ventral attention network implicated in attentional reorienting and inhibitory control [19,20,21]. Prior work showed that tDCS over rIFG improved change detection in expert SAR analysts [22], raising the question of whether similar benefits would extend to novices learning the task.

Based on the literature cited above and on our prior studies, we hypothesized the following: (1) Both the tDCS alone and tDCS combined with hf-tRNS conditions would result in better performance compared with the low-current control condition. (2) TDCS combined with hf-tRNS would produce larger behavioral benefits than tDCS alone due to the potentially additive benefits of tRNS and tDCS. (3) Differences would be observed between target detection and change detection tasks in the relative benefits provided by tDCS alone vs. tDCS combined with hf-tRNS due to possible differences in the cognitive benefits provided by each. (4) These stimulation benefits would be most pronounced in individuals with lower-baseline performance, based in part on the results of our prior studies [23]. (5) EEG measures would indicate differences associated with behavioral effects of stimulation.

## 2. Materials and Methods

### 2.1. Participants

Sixty-four (64) participants were recruited from the University of New Mexico (UNM) community. Eligibility criteria included the following: (a) normal or corrected-to-normal vision; (b) no self-reported neurological or psychiatric disorders; (c) no prior exposure to tES; and (d) no contraindications for tES or EEG. Age and gender details are shown in Table 1.

This study was conducted in accordance with the Declaration of Helsinki and approved by the Institutional Review Board of the University of New Mexico (Protocol: 1453033; Approval Date: 8 May 2019). All participants provided written informed consent and received monetary compensation or course credit.

**Table 1:** Demographics of study participants across conditions.

Variable	Total (n = 64)	tDCS (n = 20)	hf-tRNS (n = 21)	Control (n = 23)
Age, years (SD)	22.33 (6.05)	22.95 (5.62)	23.50 (5.25)	21.74 (5.25)
Male, n (%)	31 (48)	8 (40)	8 (38)	15 (65)
Female, n (%)	33 (52)	12 (60)	13 (62)	8 (35)

### 2.2. SAR Task

**Figure 1:**
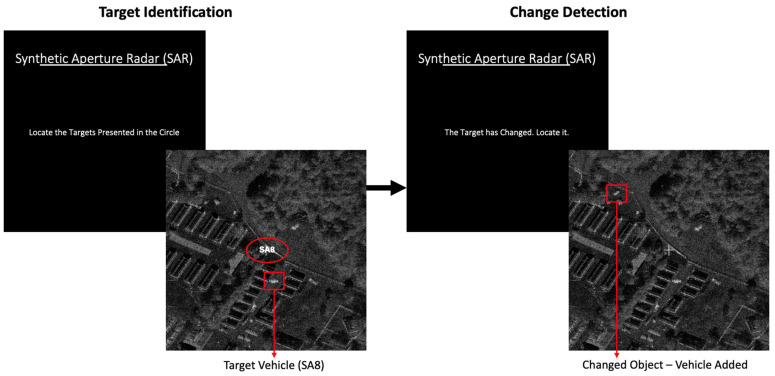
Representative screenshots of the SAR task used in both the target identification and change detection phases of each trial.

The modified SAR task required participants to: (1) identify target vehicles (SA-6 or SA-8 missile launchers) among distractors and (2) detect changes (vehicle deletion, rotation, or movement) between two image presentations (Figure 1). The original version of the SAR Task [22] had more targets (three in the original vs. two here, one target was removed to make the task easier to learn). Also, the original version included some images without targets for the target identification task, whereas all images used here contained targets and also distractors. However, some images used for the change detection task did not have any changes present. Each trial lasted up to 15 s. The task included baseline (without feedback), training (with feedback), and test sessions (without feedback), each with a total of 128 trials.

Feedback during training only indicated whether the response made by the participant was correct or incorrect. All participants completed 128 training trials, during which participants were provided feedback that identified correct responses and incorrect responses during the 15 s feedback window. Participants were allowed to make multiple selections during training. The first correct selection would end the trial and jump straight to feedback showing all incorrect and correct selections, otherwise, at the end of the 15 s response period, the feedback window would show all incorrect responses and where the correct response should have been. All participants completed the same number of trials.

### 2.3. Transcranial Electrical Stimulation (tES)

**Figure 2:**
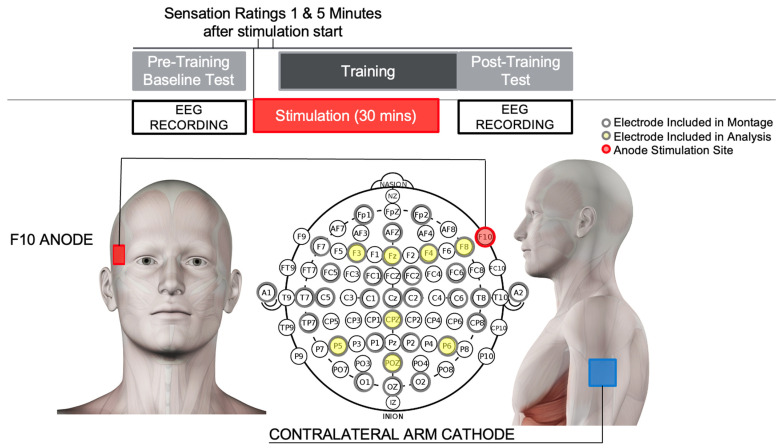
Experimental procedure and location of stimulation anode electrode (red) and cathode electrode (blue) and of EEG recording electrodes included in the analysis (yellow) relative to the 10–10 EEG montage, with recording electrodes outlined in gray. Participants first completed the pre-training baseline test (Pre-Test), followed by a training SAR task session during which stimulation (tDCS, hf-tRNS, or low-current control conditions) were administered, followed by the post-training test (Post-Test).

Stimulation was delivered using a Neuroelectrics StarStim system (Cambridge, MA, USA) via a 25 cm^2^ sponge-based cephalic electrode secured using a Neuroelectrics 64-channel neoprene EEG cap. Based on our prior tDCS studies of visual learning [10], the tDCS anode was placed over the right inferior frontal gyrus (F10), with the return (cathodal) electrode placed extracephalically over the left triceps (Figure 2).

The following three conditions were used during the 30 min training session with 30 s ramp up before stimulation and 30 s ramp down to 0 mA at the end:

1. tDCS condition: 2.0 mA DC current alone.
2. hf-tRNS condition: random noise (100–500 Hz, ±0.18 mA) combined with a 1.8 mA DC offset.
3. Low-current control condition: 0.1 mA DC current alone.

The hf-tRNS parameters were selected to maintain a total maximum current amplitude of approximately 2.0 mA (1.8 mA DC + 0.18 mA peak noise) to match the maximum current amplitude of the tDCS alone condition, while incorporating high-frequency noise intended to enhance plasticity via stochastic resonance. The combined tDCS and hf-tRNS protocol was chosen based on the prior literature suggesting that combined DC and high-frequency noise stimulation may produce synergistic effects on cortical excitability [17,18]. Participants were assigned to stimulation conditions using simple randomization. The study was single-blind.

The low-current control condition (0.1 mA) was selected to maintain blinding while remaining below the threshold for meaningful neuromodulatory effects. Prior work has demonstrated that currents below ~0.2 mA in humans do not alter cortical excitability sufficiently enough to change behavior [19]. This method has been used in many of our previous studies [10,11], and we find that it maintains blinding well, especially in participants who have no prior experience with tES such as in the present study. Although sensation ratings differed across groups, forced-choice guessing did not exceed chance accuracy, suggesting effective conditional blinding.

### 2.4. Randomization, Blinding, and Sensation Ratings

Participants were randomly assigned to one of the three tES conditions. Cutaneous sensations were assessed immediately after stimulation onset and again after 5 min of stimulation. Blinding integrity was assessed at the completion of the task. Although group-level differences in reported sensations (e.g., itching, heat, tingling) were observed between stimulation conditions, these differences did not translate into a reliable ability to correctly identify stimulation condition.

Forced-choice responses on blinding questions indicated no significant relationship between stimulation group and participant guess (r = 0.122, *p* = 0.348), and there were no significant predictive effects of sensation ratings on behavioral outcomes. Thus, despite differences in subjective experience, conditional blinding was maintained across groups.

### 2.5. EEG Acquisition and Analysis

EEG was recorded using a Neuroelectrics (Cambridge, MA, USA) StarStim system with a 64-channel neoprene EEG cap from 30 scalp electrodes during the pre-training baseline test (Pre-Test) vs. post-training test (Post-Test) periods. EEG was not collected during training due to contamination from the TES stimulation. Sampling rate was set to 500 Hz.

For analysis, after preprocessing (filtering, artifact removal and ICA) using EEGLAB (Swartz Center for Computational Neuroscience University of California, San Diego, La Jolla, CA, USA) v14.1.2b [24] via custom scripts developed in MATLAB R2019a [25] (MathWorks, Natick, MA, USA), power spectral analysis was performed for theta (4–7 Hz), alpha (8–12 Hz), and beta (13–30 Hz) bands. Regions of interest included frontal (Fz, F3, F4, F8), central (CPz), and parietal–occipital (POz, P5, P6) electrodes.

#### Statistical Analysis

Accuracy change scores for pre-training baseline tests (Pre-Test) vs. post-training tests (Post-Test) were analyzed via ANCOVA with baseline accuracy as covariate and the stimulation condition (tDCS, hf-tRNS, low-current control) as the between-subjects factor. Primary analyses were conducted separately for overall accuracy, target identification, and change detection. Pre-specified moderation analyses examined baseline performance (low vs. high) by median split to contextualize stimulation effects relative to performance. EEG analyses focused on planned regions; exploratory correlations between EEG power changes and accuracy changes were computed within conditions. The significance threshold was α = 0.05 with Šidák correction for multiple comparisons; effect sizes are reported as partial eta-squared (η*p*^2^).

## 3. Results

### 3.1. Participant Characteristics and Blinding

Complete behavioral data were available for 64 participants. Sensation ratings indicated comparable perceived intensity across conditions, consistent with preserved blinding (Table 2).

### 3.2. Behavioral Performance

#### 3.2.1. Overall Accuracy

The omnibus ANCOVA on overall accuracy change for both target identification and change detection showed a marginal but non-significant effect of condition (*F*(2,60) = 2.56, *p* = 0.086, η*p*^2^ = 0.079; Table 3). Pairwise comparisons showed tDCS produced significantly greater improvement than the low-current control condition (MD = 0.060, *p* = 0.042), while hf-tRNS showed a marginal but non-significant improvement compared with the low-current control condition (MD = 0.055, *p* = 0.079).

#### 3.2.2. Target Identification

**Figure 3:**
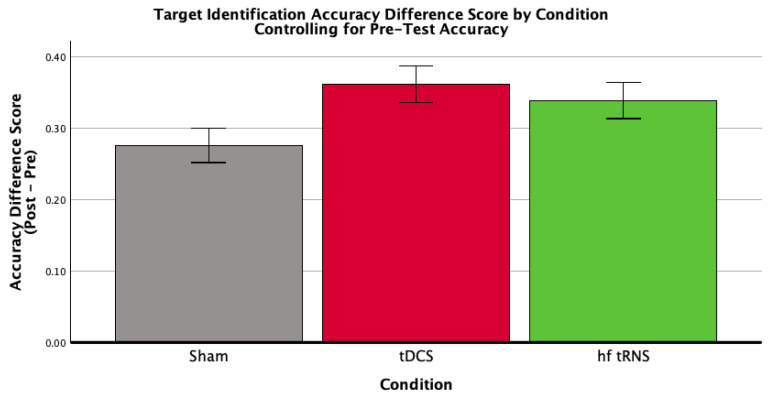
Mean accuracy difference scores (Post-Test minus Pre-Test) for each stimulation condition (Sham Control, tDCS, hf-tRNS), controlling for baseline accuracy. Error bars represent ±1 standard error. Baseline accuracy was evaluated at a mean value of 0.322. This figure depicts the results of a univariate ANCOVA examining the effect of stimulation condition on the change in target identification accuracy. The analysis revealed a significant effect of stimulation condition, *F*(2,60) = 3.27, *p* = 0.045, η*p*^2^ = 0.098.

Stimulation significantly improved target identification accuracy (*F*(2,60) = 3.27, *p* = 0.045, η*p*^2^ = 0.098; Figure 3; Table 4). Planned comparisons indicated benefits for tDCS relative to low-current control; descriptive and contrast statistics are provided in Table 4. Effects were specific to target identification, as no condition effect emerged for change detection (Section 3.2.3).

#### 3.2.3. Change Detection

**Figure 4:**
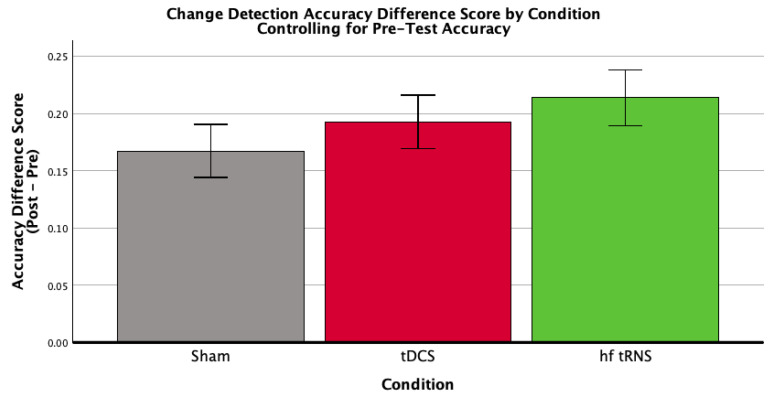
Mean accuracy difference scores (Post-Test minus Pre-Test) for each stimulation condition (Sham Control, tDCS, hf-tRNS) for change detection accuracy, controlling for baseline change detection accuracy. Error bars represent ±1 standard error. Baseline accuracy was evaluated at a mean value of 0.375. This figure depicts the results of a univariate ANCOVA examining the effect of stimulation condition on the change in change detection accuracy. The analysis revealed no significant effect of stimulation condition, *F*(2,60) = 0.87, *p* = 0.424, η*p*^2^ = 0.028.

For change detection accuracy change, the ANCOVA revealed no significant effect of condition (*F*(2,60) = 0.87, *p* = 0.424, η*p*^2^ = 0.028; Figure 4; Table 5).

### 3.3. Baseline Performance Moderation

**Figure 5:**
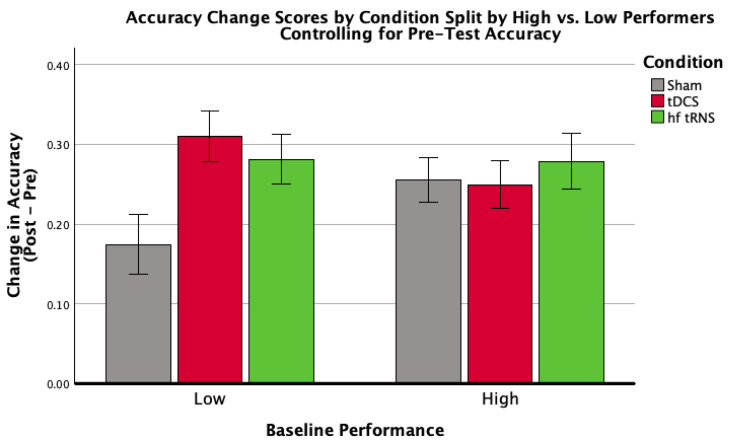
Mean accuracy difference scores (Pre-Test vs. Post-Test) for each stimulation condition (Sham Control, tDCS, hf-tRNS) and performance group (low, high), controlling for baseline overall accuracy. Error bars represent ±1 standard error. This figure illustrates the interaction effect between stimulation condition and baseline performance on the change in overall accuracy. The analysis revealed a marginal interaction effect, *F*(2,58) = 3.03, *p* = 0.056, η*p*^2^ = 0.096.

**Figure 6:**
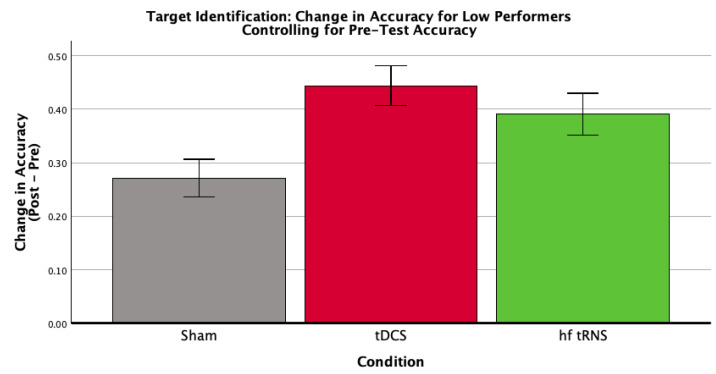
Mean target identification accuracy difference scores for each stimulation condition (Sham Control, tDCS, hf-tRNS) within the low-baseline performance group, controlling for baseline target identification accuracy. Error bars represent ±1 standard error. Baseline target identification accuracy was evaluated at a mean value of 0.256. This figure depicts the results of a univariate ANCOVA examining the effect of stimulation condition on the change in target identification accuracy within the low-performing group. The analysis revealed a significant effect of stimulation condition, *F*(2,26) = 5.99, *p* = 0.007, η*p*^2^ = 0.315.

A pre-specified analysis examined whether condition effects varied with baseline performance (low vs. high). The condition × baseline interaction was marginal but non-significant (*F*(2,58) = 3.03, *p* = 0.056). Within low-baseline performers, condition effects were significant for overall (Figure 5) and target identification accuracy (Figure 6; Table 6, Table 7 and Table 8), consistent with larger gains among initially lower performers. No significant condition effects were observed among high-baseline performers.

### 3.4. EEG Measures

EEG analysis included a total of n = 56 participants after removing 8 participants due to data quality issues identified during preprocessing. Reasons for exclusion included missing or corrupt session recordings (either Pre-Test or Post-Test) or missing event triggers. The excluded participants were distributed across the stimulation conditions as follows: two in the tDCS group, three in the hf-tRNS group, and three in the sham group. This left the final group sizes as n = 18 for tDCS, n = 19 for hf-tRNS, and n = 21 for sham.

**Figure 7:**
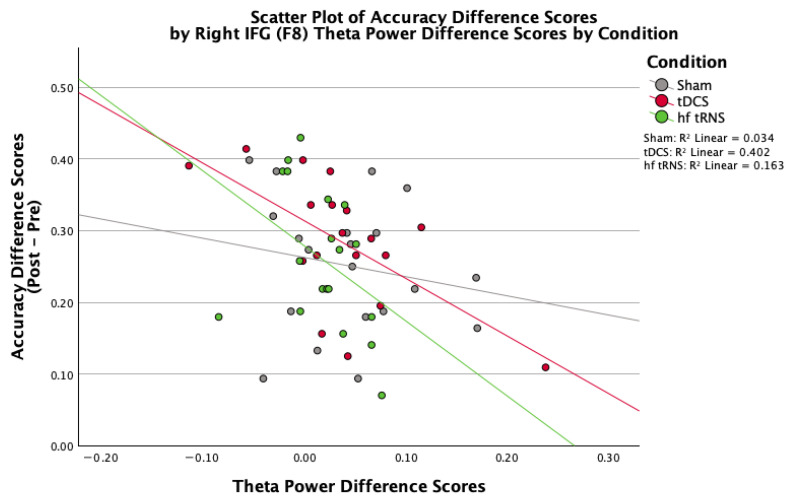
Relationship between change in F8 theta power and change in session level accuracy over the rIFG (collapsed across trial types) for each stimulation condition (control, tDCS, hf-tRNS). Change scores were calculated as Post-Test minus Pre-Test values. Each dot represents a single participant.

While group-level analyses revealed limited significant effects, correlational analyses within the tDCS group showed that participants with greater decreases in frontal (F8, under the stimulating electrode) theta power (Post-Test minus Pre-Test) were associated with larger accuracy gains (r = −0.634, *p* = 0.005; Figure 7). This significant negative correlation indicates that reduced frontal theta activity was associated with improved task performance, potentially reflecting more efficient cognitive processing following tDCS. No consistent condition effects were observed for theta/beta ratios at midline or left frontal sites. There were no significant correlations between changes in theta, alpha, or beta power at F8 and changes in accuracy in the hf-tRNS condition (all *p*s > 0.086).

## 4. Discussion

In this study, we examined the relative effects of tDCS alone and tDCS combined with hf-tRNS on performance and EEG in the SAR visual learning task for target and change detection, and differences in performance between high and low performers at baseline. We found that tDCS over rIFG selectively enhanced target identification accuracy in a complex visual search task in novices unfamiliar with the task before training. This result agrees with our previous studies using different learning tasks [10,11] and results from other laboratories in experts using a similar task [22]. Also, we found that the effects of tDCS were most pronounced among participants with lower-baseline performance before training. This agrees with prior studies showing a similar relationship between performance and stimulation effects [23]. The finding that low-baseline performers receiving tDCS surpassed even high-baseline performers post-stimulation suggests genuine facilitation of learning processes rather than mere reduction in performance variability.

The differential effects on target identification versus change detection diverge from prior findings obtained from expert analysts rather than novices [22], potentially reflecting differences in cognitive strategies between novices and experts. While experts may benefit from enhanced sustained attention supporting change detection, novices appear to gain more from improved perceptual discrimination and learning processes underlying target identification. In addition, the tDCS protocol used here was initially developed as a means to improve novice learning to more quickly reach an expert level [10]. While it has been shown to improve learning in some experts as in the prior study that this experimentwas based upon [22], it may be that it generally works better for novices than experts given that is was developed for use in novices. Indeed, our original study [10] suggested that left rather than right inferior frontal stimulation may be more effective for enhancing learning and performance in experts, based on the imaging data obtained in that study.

The selective enhancement of target identification over change detection may reflect distinct cognitive mechanisms underlying these task components. For instance, target identification primarily engages perceptual template matching and rapid attentional orienting processes, which may be more directly modulated by rIFG stimulation given this region’s role in ventral attention network function. By contrast, change detection requires sustained visual working memory maintenance and cross-temporal comparison processes that may depend more heavily on dorsal frontoparietal networks not directly targeted by our stimulation montage [26]. This dissociation suggests that tES effects on complex visual search tasks may be component-specific rather than producing global improvements in visual attention. In addition, it is well known that change detection and object identification perception processes require different but overlapping brain networks. It therefore follows that a tDCS protocol that was designed to enhance learning of target identification would be more effective for other target identification tasks than for change detection tasks. The findings of previous studies showing some benefit for change detection may be related to the overlap between the two perceptual systems. Indeed, in the current study there was some increase in performance for both tDCS and tDCS combined with hf-tRNS vs. control for change detection, but neither was statistically significant.

The hf-tRNS protocol’s lower DC offset amplitude (1.8 mA), which was 90% of the amplitude used in the tDCS condition (2.0 mA), complicated the comparison of tDCS- and tRNS-specific effects. This parameter selection was intentional to maintain a total maximum current amplitude of 2.0 mA or below across both active conditions while incorporating the noise component. However, this design makes it difficult to definitively separate the contributions of DC current from stochastic noise effects. The similarity between the performance improvement ratios for tDCS (119.32%) versus hf-tRNS (105.46%) conditions (ratio = 1.13) and the ratio of DC current amplitudes (2.0/1.8, ratio = 1.11) suggests that the application of high-frequency noise may not have produced additional effects beyond those attributable to the linear effects of the DC component alone. The lack of significant effects in the hf-tRNS condition may therefore be related to linear effects of DC current strength on behavior alone.

Finally, the correlation between decreased frontal theta and improved performance in the tDCS group aligns with the literature suggesting reduced theta may reflect more efficient cognitive processing [27]. In other words, the cognitive enhancement provided by tDCS leads to a reduced demand for the endogenous facilitation indicated by power in the theta band. However, the limited group-level EEG effects highlight challenges in identifying reliable neurophysiological markers of tES effects.

### 4.1. Limitations

Key limitations include the following: (1) Novice participants were used here, limiting comparisons with prior SAR task studies using tES with experts [22]. This also limits generalizability to active operational contexts, where experienced analysts would typically be present, and instead focuses on potential applications for enhanced performance during initial training. (2) This study did not include measures of attention or other processes to clarify what cognitive benefits of tDCS led to improvement in target detection, and not to change detection. (3) Technical issues in data collection reduced the sample size for the EEG analyses from 64 to 56, thus reducing power to observe changes in factors aside from theta power. (4) TDCS amplitude was limited to 1.8 mA for the hf-tRNS condition in order to maintain the maximum current at 2.0 mA between conditions. However, this made comparison with the tDCS alone condition more difficult, given that the mean current was higher in the tDCS alone condition. It appears from the results of the present study that mean current may be more important for equating tES conditions than maximum current.

### 4.2. Future Directions

Future research could examine a number of topics suggested by the present study: (1) tES effects could be studied in a range of novice to expert populations to examine how tES effects change with expertise. In addition, anodal electrode placement from right to left inferior frontal gyrus could show whether experts benefit more from left than right stimulation. (2) TDCS and tRNS parameters could be varied independently and systematically to isolate the contribution of noise and DC effects on cognition and performance. Also, using the identical DC current strength in both the tDCS and hf-tRNS conditions would be more beneficial for isolating the separate effects of tRNS. (3) Attention-specific and other cognitive tasks (such as working memory, long-term memory, and perceptual discrimination tasks) could be employed to further clarify the specific cognitive benefits of these tES protocols, and to determine whether improved performance during target detection was due to improved learning during training, or improved attention during testing, among other possibilities. It also may help to explain the greater benefit observed for target detection relative to change detection. (4) Using higher-resolution neurophysiological recordings, including HD-EEG and MEG, and in a larger group of participants offering greater statistical power, would help to better understand the mechanisms of neural changes evoked by tES. (5) Investigating individual differences predicting tES responsiveness in addition to baseline performance used here would help to examine the question of whether baseline performance itself is important for sensitivity to tDCS effects, or whether there is some other factor leading to both lower-baseline performance and greater effects of tDCS?

## 5. Conclusions

TDCS over rIFG enhanced target identification accuracy in novice participants performing a complex visual search task, with effects greater in low-baseline compared with high-baseline performers. The hf-tRNS condition produced no significant effects on performance, with differences in mean effect size approximately proportional to differences in DC amplitude between these conditions, with little or no additional benefits of added hf-tRNS. These findings support tDCS as a potential tool for facilitating specific aspects of perceptual learning, though effects appear task-component-specific, with a greater benefit for target detection than change detection in the novice population studied here. The finding of decreased theta EEG amplitude in participants who performed better in the target detection test task in the tDCS group suggests that tDCS-related cognitive facilitation reduced the compensatory theta activity typically associated with task difficulty. Further research is needed to optimize stimulation parameters and clarify mechanisms before the use of tDCS in translational real-world operational training contexts.

## Figures and Tables

**Table 2:** Mean and standard deviation of sensation ratings (tingling, itching, heat) at two time points during stimulation. Despite group-level differences in sensation intensity, blinding was maintained across conditions.

Condition	Tingling T1	Tingling T2	Itching T1	Itching T2	Heat T1	Heat T2
Control	1.29 (±1.68)	0.76 (±1.30)	1.24 (±1.76)	0.71 (±1.23)	0.24 (±0.54)	0.29 (±0.78)
tDCS	3.10 (±1.94)	1.55 (±1.57)	3.05 (±1.91)	2.05 (±1.70)	2.45 (±2.21)	1.10 (±1.25)
hf-tRNS	2.84 (±1.89)	1.63 (±1.54)	3.21 (±1.75)	2.11 (±1.20)	1.63 (±1.71)	0.95 (±1.27)

**Table 3:** Descriptive statistics for accuracy by condition.

Condition	Mean Pre-Test Accuracy (SD)	Mean Post-Test Accuracy (SD)	Mean Difference Score (SD)	n
Control	0.313 (0.097)	0.551 (0.142)	0.238 (0.117)	23
tDCS	0.340 (0.062)	0.625 (0.055)	0.284 (0.086)	20
hf-tRNS	0.394 (0.079)	0.646 (0.079)	0.251 (0.099)	21
Total	0.348 (0.087)	0.605 (0.108)	0.257 (0.102)	64

**Table 4:** Descriptive statistics for target identification accuracy by condition.

Condition	Mean Pre-Test Accuracy (SD)	Mean Post-Test Accuracy (SD)	Mean Difference Score (SD)	n
Control	0.307 (0.119)	0.597 (0.139)	0.290 (0.160)	23
tDCS	0.307 (0.096)	0.683 (0.093)	0.376 (0.135)	20
hf-tRNS	0.353 (0.126)	0.662 (0.099)	0.310 (0.172)	21
Total	0.322 (0.115)	0.645 (0.118)	0.323 (0.159)	64

**Table 5:** Descriptive statistics for change detection accuracy by condition.

Condition	Mean Pre-Test Accuracy (SD)	Mean Post-Test Accuracy (SD)	Mean Difference Score (SD)	n
Control	0.319 (0.119)	0.505 (0.162)	0.185 (0.117)	23
tDCS	0.373 (0.072)	0.566 (0.103)	0.193 (0.112)	20
hf-tRNS	0.436 (0.102)	0.630 (0.090)	0.193 (0.098)	21
Total	0.375 (0.110)	0.565 (0.133)	0.190 (0.108)	64

**Table 6:** Descriptive statistics for low-baseline performance group overall accuracy by condition.

Condition	Mean Pre-Test Accuracy (SD)	Mean Post-Test Accuracy (SD)	Mean Difference Score (SD)	n
Control	0.236 (0.041)	0.479 (0.139)	0.243 (0.125)	11
tDCS	0.296 (0.045)	0.638 (0.046)	0.341 (0.052)	10
hf-tRNS	0.322 (0.052)	0.619 (0.086)	0.297 (0.120)	9
Total	0.282 (0.058)	0.574 (0.122)	0.292 (0.109)	30

**Table 7:** Descriptive statistics for low-baseline performance group target identification accuracy by condition.

Condition	Mean Pre-Test Accuracy (SD)	Mean Post-Test Accuracy (SD)	Mean Difference Score (SD)	n
Control	0.251 (0.080)	0.528 (0.132)	0.277 (0.167)	11
tDCS	0.256 (0.081)	0.700 (0.112)	0.444 (0.104)	10
hf-tRNS	0.262 (0.120)	0.646 (0.097)	0.384 (0.196)	9
Total	0.256 (0.091)	0.621 (0.135)	0.365 (0.169)	30

**Table 8:** Descriptive statistics for low-baseline performance group change detection accuracy by condition.

Condition	Mean Pre-Test Accuracy (SD)	Mean Post-Test Accuracy (SD)	Mean Difference Score (SD)	n
Control	0.220 (0.085)	0.429 (0.165)	0.209 (0.118)	11
tDCS	0.336 (0.059)	0.575 (0.114)	0.239 (0.127)	10
hf-tRNS	0.382 (0.088)	0.592 (0.102)	0.210 (0.115)	9
Total	0.307 (0.103)	0.527 (0.148)	0.219 (0.117)	30

## Data Availability

The raw data supporting the conclusions of this article will be made available by the authors on request once ongoing efforts to win patents related to this work are concluded.

## Abbreviations

The following abbreviations are used in this manuscript:

tES	Transcranial electrical stimulation
tDCS	Transcranial direct current stimulation
hf-tRNS	High-frequency transcranial random noise stimulation
rIFG	Right inferior frontal gyrus
SAR	Synthetic aperture radar
IPS	Intraparietal sulcus
FEF	Frontal eye fields
DLPFC	Dorsolateral prefrontal cortex
UNM	University of New Mexico
